# Nursing privilege: A concept analysis

**DOI:** 10.1002/nop2.2120

**Published:** 2024-03-21

**Authors:** Ahmad A. Abujaber, Abdulqadir J. Nashwan

**Affiliations:** ^1^ Nursing Department Hamad Medical Corporation Doha Qatar

**Keywords:** competency, concept analysis, nursing, privilege, scope of practice

## Abstract

**Aim:**

The study aimed to provide a comprehensive concept analysis of nursing privileges by elucidating its meaning and implications within the healthcare context.

**Design:**

A concept analysis paper.

**Methods:**

A comprehensive literature review was conducted from nursing and healthcare databases, professional nursing organizations, and regulatory bodies. Documents reviewed include research studies, policy documents and professional guidelines. The study employed Walker and Avant's eight‐step method of concept analysis. This involved identifying the uses of the concept, its underlying attributes and referents, and constructing model, borderline, related and contrary cases. The antecedents, consequences and empirical referents of nursing privileges were also determined.

**Results:**

The analysis uncovered vital attributes defining nursing privileges, encompassing professional authority, autonomy, access to resources, information, influence, decision‐making power, respect and recognition. Additionally, antecedents and consequences of nursing privilege were identified, spanning development and resource access, as well as professional satisfaction and enhanced patient care.

**Patient or Public Contribution:**

No patient or public contribution.

## 1 INTRODUCTION

Concept analysis is highly valuable due to its ability to offer contemporary theoretical and practical definitions, thereby facilitating their application in research and theory (Brush et al., 2011; Walker & Avant, 2005). It is crucial to recognize that concepts are not fixed entities; they evolve alongside acquiring new knowledge, leading to potential variations in interpretation among analysts (Walker & Avant, 2005). Consequently, concept analysis proves applicable across diverse disciplines, regardless of whether the terms in question have been in use for short or long durations or are emerging in research. However, the perception of concept analysis within the nursing scholarship community varies significantly, encompassing divergent viewpoints on its value and contribution. While some scholars have faced difficulties and expressed scepticism regarding its actual contribution, others argue that these methods fail to generate a practical, theoretical framework, deeming concept analysis as an arbitrary and vacuous exercise (Morse, 1995; Paley, 1996). The aim of this work is to foster a comprehensive comprehension of nursing privilege, encompassing its various components and implications within the nursing profession. Through a thorough exploration of this concept, we intend to contribute to the ongoing discussions surrounding privilege and power dynamics in healthcare.

## 2 BACKGROUND

The term ‘privilege’ often denotes the unearned advantages and benefits that certain individuals or groups possess in society, often attributed to social status, wealth, race, gender or education (Black & Stone, 2005). Stemming from critical social justice literature, the concept of privilege emerged to examine the inherent advantages and disadvantages experienced by individuals based on their social identities (Goforth & Pham, 2023). As time progressed, the concept of privilege underwent evolution and redefinition, giving rise to a new area of theory and research (Bergkamp et al., 2022). In the realm of healthcare, clinical privilege pertains to the authorization granted by hospitals or healthcare institutions to healthcare providers, enabling them to deliver specific care services within well‐defined limits, contingent upon their credentials and qualifications (Jansen, 2009). Undertaking a concept analysis of privilege becomes crucial to elucidate its defining attributes, antecedents and consequences. Moreover, such analysis aids in developing interventions and strategies to address and mitigate the effects of privilege in both healthcare and society.

**FIGURE 1:**
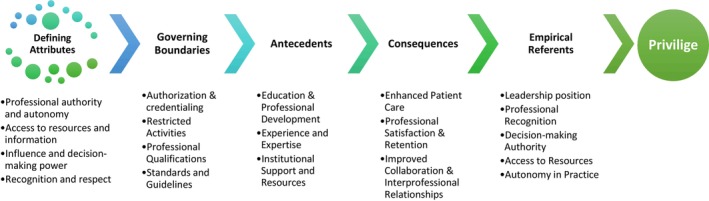
Summary of the key elements of the concept analysis of nursing privilege.

In this paper, the concept of privilege and clinical nursing privilege will be analysed using the Walker and Avant method (Walker & Avant, 2005). This method was chosen for its simplicity and direct approach. The methodology comprises 8 key steps, namely: selecting a concept, determining the aim of the analysis, discerning the defining attributes of the concept, exploring various uses of the concept, identifying a model case, identifying borderline, related, and contrary cases, identifying antecedents and consequences and, finally, establishing an empirical referent. These steps provide a structured framework for comprehensively analysing the concept under investigation. Figure 1 encapsulates the fundamental components unearthed in the concept analysis of nursing privilege.

## 3 LITERATURE SEARCH

In conducting a literature review on ‘Nursing Privilege’, a systematic search strategy was employed using the PubMed, Scopus and CINAHL Ultimate databases. The search was tailored to include a combination of specific MeSH (Medical Subject Headings) terms and the keyword ‘Privilege’. The selected MeSH terms were: ‘Nursing’, ‘Nursing Care’, ‘Nursing Services’, ‘Nursing Staff’, ‘Nursing Evaluation Research’, ‘Nursing Theory’, ‘Nurse‐Patient Relations’, and ‘Specialties, Nursing’. These terms were chosen to encompass a broad range of topics within the field of nursing that might intersect with the concept of privilege. The search strategy involved using Boolean operators to expand the search, structured as follows for PubMed: ((((((((Nursing) OR (Nursing Care)) OR (Nursing Services)) OR (Nursing Staff)) OR (Nursing Evaluation Research)) OR (Nursing Theory)) OR (Nurse‐Patient Relations)) OR (Specialties, Nursing)) AND (Privilege).

The search in PubMed/Medline revealed 1035 articles, while a parallel search in Scopus revealed 645 documents. Additionally, a search in CINAHL Ultimate revealed 81 documents. The resultant articles from all three databases were then screened based on relevance to the topic, publication date and the presence of peer‐review, to ensure the inclusion of only high‐quality and pertinent literature. The final selection of articles was subjected to a detailed review to extract data on the nature and attributes of privilege in nursing by scanning the abstracts and full texts as applicable. This process of synthesis aimed to present a comprehensive overview of the current state of knowledge on the topic, identifying key themes, trends and gaps in the existing literature.

## 4 USES OF THE CONCEPT

The term ‘privilege’ finds widespread usage in various contexts, generally referring to granting immunity or specific rights to individuals, groups or entities based on specific characteristics (Merriam‐Webster Dictionary, 2005). In the realm of law and legal matters, privilege is defined as a legal rule that safeguards communications within specific relationships, preventing their compelled disclosure in court proceedings. One enduring example of such privilege, applicable in all legal settings, is the attorney–client privilege (Plush, 2006). Privileges are typically established by law and can vary depending on the jurisdiction and the context in which they are invoked (Good et al., 2004). These privileges encompass a range of categories, including attorney–client privilege, doctor‐patient privilege, spousal privilege, executive privilege or diplomatic privilege. Each privilege protects confidential or sensitive information, promotes open communication or safeguards specific interests within the legal framework. It is crucial to note that privileges are subject to distinct legal requirements and limitations, and their scope and application may vary based on the jurisdiction and specific circumstances involved (Bower & Bilsland, 2016).

In the context of social justice, the term ‘privilege’ often entails the recognition of discrimination, racism, oppression and injustice, acknowledging that certain individuals may benefit from advantages in society solely due to characteristics such as their race, gender, socioeconomic status, sexual orientation or ability, while others may encounter systemic disadvantages (Goforth & Pham, 2023). The application of the term ‘privilege’ in the realm of social justice encompasses various uses, including:

- White privilege denotes the societal advantages and privileges afforded to individuals based on their race, specifically white individuals. It encompasses a range of benefits, including enhanced access to education, employment opportunities and the presumption of innocence within the criminal justice system (Jason, 2020; McIntosh, 2018).
- Male privilege acknowledges the advantages and benefits those men receive within society solely based on their gender. These advantages include higher wages, greater representation in leadership roles and freedom from gender‐based violence and discrimination (McIntosh, 2018).
- The class privilege recognizes the inherent advantages that individuals from higher socioeconomic classes possess, including privileged access to quality education, healthcare and economic opportunities, in contrast to individuals from lower socioeconomic backgrounds (Barak et al., 2010).
- Able‐bodied privilege acknowledges the advantages that individuals without disabilities enjoy in terms of accessibility, mobility and social inclusion, in contrast to individuals with disabilities (Palombi, 2013).

In the realm of academia and education, the examination of privilege serves to comprehend disparities in access and academic achievement. Within this context, privilege can take the form of advantages granted to individuals based on factors like socioeconomic background, educational opportunities or parental support. Analysing privilege in education aids in the identification of barriers that impede educational attainment for marginalized groups, and it informs endeavours to foster equitable access and opportunities for all students (Blanchett, 2006; Carnevale & Strohl, 2013).

In the medical field, the term ‘privilege’ pertains to physicians' advantages and authority. It encompasses their decision‐making power, the ability to prescribe medications and their control over patient care plans. Physician privilege also entails the trust and respect bestowed upon doctors by patients and other healthcare professionals (Hatem & Ferrara, 2001).

Building upon the broad concept of privilege, a more specific subset emerges, known as professional or clinical privilege. This notion transcends the realm of physicians, encompassing a diverse range of healthcare professionals, including but not limited to nurses, pharmacists and allied health practitioners. This recognition acknowledges their specialized knowledge, skills and expertise, which grant them specific rights and responsibilities in patient care (Jansen, 2009). According to Law Insider, clinical privilege refers to the advantage of being authorized to perform specific nursing activities or procedures restricted to licensed healthcare providers (Insider, 2023). However, it is important to note that there currently needs to be a universally defined concept of nursing privilege, and there is a lack of prior nursing literature specifically addressing this term. The utilization of the term ‘nursing privilege’ in the nursing field originated from the credentialing process employed to licence and authorize medical practitioners and professionals to practice their respective roles.

While dictionaries clearly define the term ‘privilege’, its direct application to the nursing profession may not always yield meaningful results. Nursing regulatory bodies worldwide grant nurses authorizations to deliver patient care based on specific qualifications and requirements. However, it is worth noting that the term ‘nursing privilege’ is not commonly utilized, with ‘clinical privilege’ or ‘medical privilege’ being more prevalent in the field.

## 5 DEFINING ATTRIBUTES

Defining attributes are the characteristics of a concept that appear repeatedly in the literature and are consistently present when the concept occurs (Walker & Avant, 2005).

This section aims to recognize the essential qualities that are necessary for the establishment of nursing privilege. A key aspect of nursing privilege is the authorization to practice within certain boundaries in accordance with the rules and regulations of the governing body. The authorization is determined based on certain qualifications that are obtained from accredited institutes such as universities and professional certification bodies and, finally, the governance structure within which ethical guidelines, professional standards and regulatory policies govern the nurse practice.

The following four boundaries determine the defining attributes of privilege in nursing:

- Authorization and credentialing: Privilege involves granting specific rights and permissions to healthcare providers by regulatory bodies.
- Restricted Activities: Privilege limits certain nursing activities or procedures to licensed and qualified professionals.
- Professional Qualifications: Privilege is linked to the educational background, certifications and training that demonstrates competence in performing restricted activities.
- Standards and Guidelines: Privilege is governed by professional standards, ethical guidelines and regulatory policies that ensure safe and effective practice.

Based on the above four boundaries, here are the defining attributes of nursing privilege;

- Professional authority and autonomy: Nurses have a distinct professional role and are entrusted with a level of authority and autonomy within the healthcare system. They possess specialized knowledge and skills that allow them to make independent decisions regarding patient care. This authority and autonomy contribute to the privilege nurses may experience in their professional practice. For example, nurses may have the authority to prescribe medications, develop care plans and lead healthcare teams (Hood, 2013).
- Access to resources and information: Nurses often have access to valuable resources, such as education, training and healthcare information, which can contribute to their privileged position. They could continuously update their knowledge and skills through professional development programmes, conferences and research publications. This access to resources empowers nurses to provide high‐quality care and positions them as knowledgeable professionals within the healthcare system (Angela, 2020; Blais, 2015).
- Influence and decision‐making power: Nurses may possess the ability to influence decisions related to patient care, policies and healthcare delivery. They are involved in various aspects of decision‐making, ranging from individual patient care decisions to participating in healthcare committees and policy development. Nurses' input and expertise are valued and considered when making decisions that impact patient outcomes and nursing practice (Jesus & Julia, 2020; Yoo et al., 2019)
- Recognition and respect: Nursing privilege involves the acknowledgement and respect given to nurses by colleagues, patients and the broader society. Nurses are often regarded as trusted healthcare professionals and play a vital role in the well‐being of individuals and communities. This recognition and respect contribute to nurses' professional status and influence their interactions with other healthcare providers, patients and the general public (Sepasi et al., 2016).

## 6 MODEL CASE

A model case serves as a practical and comprehensive example that incorporates all the defining attributes of a concept (Brush et al., 2011). It serves as an exemplar, providing a tangible representation of how the concept of nursing privilege can manifest in a real‐world scenario. In the following section, we will construct a model case to demonstrate a scenario that encompasses the defining attributes of nursing privilege:

### 6.1 Model case

Aya exemplifies an experienced nurse who holds a leadership position in a prominent hospital. Through her diligent efforts, she has advanced her career and garnered the admiration of her colleagues and superiors. Aya's commitment to professional development is evident, as she has pursued advanced education and training, culminating in attaining a master's degree in Nursing Administration. As a result of her expertise, she is entrusted with the pivotal role of managing a team of nurses, formulating care protocols and making critical decisions that significantly impact patient care.

In her capacity as a nursing leader, Aya enjoys professional authority and autonomy. She can make crucial decisions about staffing, resource allocation and workflow management within her department. This level of authority empowers her to advocate for her team and ensure the effective delivery of patient care, thereby fostering a supportive and conducive healthcare environment. Aya's model case embodies the defining attributes of nursing privilege, showcasing how expertise, leadership and decision‐making authority converge to elevate the nursing practice and enhance patient outcomes.

In addition to her authority, Aya benefits from various resources and information that amplifies her privileged position. She actively participates in conferences, workshops and continuing education programs to remain updated on the latest evidence‐based practices. This knowledge equips Aya with the capacity to implement innovative strategies that enhance patient outcomes and advance nursing practice within her department.

Aya's sphere of influence extends beyond her immediate team. She actively engages in hospital committees, collaborating with fellow healthcare professionals to shape policies and improve healthcare delivery. Her contributions are highly regarded, and she holds a seat at the decision‐making table where choices affecting patient care are deliberated upon.

As a respected nursing leader, Aya garners recognition and respect from her colleagues, patients, and the wider healthcare community. Her expertise and contributions are acknowledged, positioning her as a role model for aspiring nurses. Aya's privilege as a nurse becomes evident through the trust and admiration she receives, both within and beyond her organization.

This model case exemplifies how nursing privilege materializes through professional authority and autonomy, access to resources and information, influence, decision‐making power, recognition and respect. It portrays a nurse who has adeptly navigated the nursing profession, utilizing her position to positively impact patient care and the nursing profession as a whole.

## 7 ADDITIONAL CASES

Analysing cases that are dissimilar or highly similar to the concept of interest is an additional approach to refining the defining attributes during a concept analysis (Brush et al., 2011; Walker & Avant, 2005). Here, we present examples of borderline, contrary and related cases to aid in this process. The cases discussed in our manuscript are not direct, real‐world cases, but rather they are derived from the collective professional experience of the authors. These cases represent composites of various scenarios and experiences we have encountered in our nursing practice, adapted and generalized to illustrate the concept of Nursing Privilege more effectively.

### 7.1 Borderline case

Borderline cases are scenarios that possess some, but not all, of the defining attributes of nursing privilege (Alannah et al., 2020). These cases help to further clarify the concept by highlighting situations that may be on the boundary or exhibit partial aspects of nursing privilege. Here is an example of a borderline case:

#### 7.1.1 Case

Yara is a nurse working in a rural healthcare facility. While she does not hold a leadership position or have statistically significant decision‐making power, she is dedicated to providing the best possible care to her patients. Yara actively seeks opportunities for professional development, attends relevant workshops and conferences and keeps herself updated with the latest evidence‐based practices. Although she has a different level of access to resources and information than nurses in larger institutions, she takes advantage of online resources and networks with colleagues to enhance her knowledge and skills.

In this case, Yara exhibits some attributes of nursing privilege. She is committed to professional development, actively seeking opportunities to enhance her knowledge and skills. However, she needs more professional authority, autonomy and extensive access to resources typically associated with nursing privilege. Despite these limitations, Yara's proactive approach and dedication contribute to her ongoing growth as a nurse.

This borderline case illustrates how nursing privilege can manifest nuancedly, with individuals like Yara striving for professional development despite facing limited resources and authority. It reminds us that nursing privilege is not a one‐size‐fits‐all concept but rather can vary across different contexts and settings. It highlights the resilience and determination of individuals who navigate their professional journey with a sense of agency and ambition, even without certain privileges typically associated with the concept.

Examining such cases helps us broaden our understanding of nursing privilege and recognize the diverse ways it can be experienced. It prompts us to consider the influence of external factors, such as institutional resources and hierarchical structures, on the manifestation of nursing privilege. It also encourages us to explore ways to support and empower nurses like Yara, who exhibit dedication and a drive for professional growth, despite facing challenges in their professional environment.

### 7.2 Contrary case

A contrary case represents a scenario that contradicts or opposes the defining attributes of the concept being analysed (Alannah et al., 2020). It serves as a valuable tool to further clarify the boundaries and distinctive features of the concept, allowing for a more comprehensive understanding. Here is an example of a contrary case for the concept of nursing privilege: Karam is a nurse who works in a busy hospital. Despite having many years of experience, he feels disempowered and needs more authority in his role. He often needs help accessing necessary resources and information to provide optimal patient care. Karam’s suggestions and input are often disregarded by his colleagues and superiors, leading to a lack of recognition and respect within the healthcare team.

7.2.1

In this contrary case, Karam's experience challenges the defining attributes of nursing privilege. He needs more professional authority, autonomy and access to resources essential to nursing privilege. Furthermore, his lack of recognition and respect within the healthcare setting contradicts the idea of privilege. This case highlights the absence or negation of key attributes associated with nursing privilege, helping to delineate the concept's boundaries and clarify what it is not.

By examining this contrary case, we gain a deeper understanding of the factors that can inhibit nursing privilege and the potential barriers that exist within healthcare systems. Karam's experience highlights the importance of addressing power differentials, promoting collaborative decision‐making and providing equitable access to resources and opportunities. In addition, it reinforces the significance of recognizing and addressing instances where nursing privilege may be absent or diminished, contributing to the ongoing refinement and understanding of the concept within the nursing profession.

## 8 ANTECEDENTS AND CONSEQUENCES

Identifying the antecedents and consequences of a concept helps us understand the factors that precede or contribute to the concept's existence and the outcomes or effects that result from it (Nagel et al., 2022). In the case of nursing privilege, examining its antecedents and consequences provides valuable insights into the underlying conditions and impacts associated with this concept.

### 8.1 Antecedents of nursing privilege

Antecedents are the conditions or factors that precede or contribute to the concept of nursing privilege (Walker & Avant, 2005). They provide insight into the circumstances or requirements that enable the development or recognition of nursing privilege. Some examples of antecedents of nursing privilege may include:

- Education and Professional Development: A solid educational foundation and ongoing professional development are antecedents that can contribute to developing nursing privilege. Nurses who have received comprehensive education and continue to enhance their knowledge and skills are more likely to gain recognition, authority and autonomy within their practice (Hickey et al., 2014).
- Experience and Expertise: Accumulated experience and expertise in nursing practice are important antecedents of nursing privilege. As nurses gain more experience and develop specialized skills and knowledge in specific areas, they may be granted higher authority, responsibility and recognition within their professional roles (McHugh & Lake, 2010; Miltner et al., 2015).
- Institutional Support and Resources: Institutions prioritizing nursing practice and providing adequate support and resources create an environment conducive to nursing privilege. Access to resources, such as staffing, technology and educational opportunities, can empower nurses and enable them to fulfil their professional roles effectively (Gillet et al., 2013).

### 8.2 Consequences of nursing privilege

Consequences are the outcomes or effects that result from the concept of nursing privilege (Earvolino‐Ramirez, 2007). They illustrate the impact of nursing privilege on various aspects of nursing practice and the healthcare system. Some examples of consequences of nursing privilege may include:

- Enhanced Patient Care: Nursing privilege can lead to improved patient care outcomes. Nurses with privilege may have the authority and autonomy to make decisions that optimize patient care, implement evidence‐based practices and advocate for their patient's needs, resulting in better health outcomes and patient satisfaction (Coelho, 2020; Whitehead et al., 2019).
- Professional Satisfaction and Retention: Nursing privilege can contribute to increased professional satisfaction and retention rates among nurses. When nurses are recognized, respected and have a sense of authority and autonomy in their roles, they are more likely to feel fulfilled in their profession and remain engaged in their practice (Cartwright, 2021).
- Improved Collaboration and Interprofessional Relationships: Nursing privilege can foster positive collaboration and relationships among healthcare professionals. Nurses with privilege may be considered valuable contributors to the healthcare team, leading to enhanced teamwork, communication and cooperation with other healthcare providers (Coelho, 2020).

These examples of antecedents and consequences provide a deeper understanding of the factors that contribute to nursing privilege and the outcomes it can generate. By exploring these aspects, we can gain insights into the conditions and effects surrounding nursing privilege and its impact on nursing practice and patient care.

## 9 EMPIRICAL REFERENTS

Defining empirical referents involves identifying observable indicators or measurements representing the concept being analysed (Alannah et al., 2020). Empirical referents help to operationalize the concept and provide concrete ways to measure or assess its presence or absence (Alannah et al., 2020). Defining empirical referents allows for the objective measurement and evaluation of the concept of nursing privilege, enabling researchers and practitioners to assess its presence and impact within the nursing profession. In addition, these empirical referents provide tangible indicators that can be observed and measured, contributing to a deeper understanding of nursing privilege and its manifestations.

Empirical referents for Nursing Privilege may include:

- Leadership Positions: One empirical referent for nursing privilege could be the presence of nurses in leadership positions within healthcare organizations. This can be measured by the number or percentage of nurses holding managerial or administrative roles (Aspinall et al., 2019).
- Professional Recognition: Another empirical referent for the nursing privilege is recognizing and acknowledging nurses' expertise and contributions. This can be measured through awards, accolades or professional affiliations that signify recognition of a nurse's accomplishments and impact (Fitzgerald, 2020).
- Decision‐making Authority: The level of decision‐making authority granted to nurses can serve as an empirical referent for the nursing privilege. This can be assessed by examining the extent to which nurses are involved in making clinical, organizational or policy decisions (Haahr et al., 2020; Kemppainen et al., 2013).
- Access to Resources: The availability and accessibility of resources, such as staffing, educational opportunities, research materials and technology, can be used as empirical referents for the nursing privilege. This can be measured by assessing the availability and utilization of these resources within healthcare settings (Angela, 2020; Blais, 2015).
- Autonomy in Practice: The degree of autonomy granted to nurses in their practice can be considered an empirical referent for nursing privilege. This can be evaluated by examining the extent to which nurses have the freedom to make independent decisions and implement their professional judgement in patient care (Hood, 2013; Pursio et al., 2021).

Interestingly, our analysis revealed that there are two frequently alluded concepts significantly enhance the relevance and utility of nursing privilege: access to resources and the ability to influence. These components bolster nurse autonomy, enabling active participation in decision‐making, leadership and ultimately contribute to improved patient care and heightened professional satisfaction.

## 10 DISCUSSION

This study endeavours to dissect the concept of ‘nursing privilege’ by exploring its attributes, antecedents and consequences. The primary objective is to formulate an operational definition that is both relevant and meaningful, accounting for the diverse contexts in which this concept may be applied. Through a comprehensive analysis, we aim to reveal the intricate nature of nursing privilege and its far‐reaching implications in various healthcare settings.

Importantly, it is crucial to emphasize on the importance of recognizing nursing privilege as a dynamic and evolving concept, influenced by factors such as authority, autonomy and access to resources. It acknowledges the variations in how nursing privilege is experienced across different contexts and settings, emphasizing the need for a nuanced understanding of this concept in both academic discourse and practical healthcare applications. The suggested operational definitions are tailored to different contexts, offering nuanced perspectives for academic and operational settings.

In delineating the concept of ‘nursing privilege’ through its attributes, antecedents, and consequences, it is imperative to craft a precise operational definition. It encapsulates the identified attributes, antecedents and consequences of nursing privilege to enhance the comprehensiveness of the definition.

- Comprehensive operational definition of nursing privilege
  Nursing Privilege is a professional status in healthcare characterized by the possession of professional authority and autonomy, facilitating access to essential resources and information. This status empowers nurses with influence and decision‐making power, fostering an environment where they receive due respect and recognition. Antecedents involve robust educational qualifications and credentials, while consequences include heightened professional satisfaction and the ability to enhance patient care through informed and autonomous practice.

Nevertheless, given the complex and multi‐dimensional nature of the concept ‘nursing privilege’, we have broadened the operational definition's scope to capture and incorporate some identified central components that give the concept ‘nursing privilege’ its significance and relevance in both academic and operational settings. We pinpointed two pivotal facets: access to resources, reflected in the resource‐centric operational definition, and influence, outlined in the influence‐based operational definition. So, beside the comprehensive definition, we presented another two operational definitions tailored to the specific scope and context in which the concept ‘nursing privilege’ may be applied.

- Resource‐centric operational definition
  Nursing Privilege is the specialized status wherein nurses have exclusive access to crucial resources and information. It involves the authority to influence decisions, autonomy in practice and garnering respect. Antecedents are rooted in educational achievements, and consequences include a profound impact on patient care and professional satisfaction, particularly through resource optimization and informed decision‐making.
- Influence‐focused operational definition
  Nursing Privilege denotes a professional standing marked by influence and authority within health care. It grants autonomy, access to resources and the power to make decisions. Rooted in educational qualifications, it leads to positive consequences such as professional satisfaction and elevated patient care standards. This operational definition underscores the centrality of influence and decision‐making power in the nursing profession.

## 11 FUTURE IMPLICATIONS

In essence, a well‐defined concept of nursing privilege holds the potential to reshape nursing practice, education and policy, ultimately contributing to a more empowered and effective nursing workforce and improved patient outcomes. The comprehensive understanding and definition of the concept ‘nursing privilege’ can have several statistically significant future implications across various domains:

### 11.1 Policy development

A nuanced understanding of nursing privilege can inform the development of policies within healthcare institutions. This insight may lead to policies that foster an environment where nursing privileges are recognized, valued and utilized to enhance patient care.

### 11.2 Education and training

Educational programs for nurses can be tailored to incorporate the dimensions of nursing privilege. This includes curricula that emphasize the development of attributes associated with nursing privilege, such as autonomy, leadership and effective decision‐making.

### 11.3 Professional development

Healthcare organizations may use the insights gained to structure professional development opportunities for nurses. This could involve targeted training programs that enhance nurses' access to resources, decision‐making skills and leadership capabilities.

### 11.4 Workplace culture and recognition

Understanding nursing privilege can contribute to cultivating a positive workplace culture that recognizes and respects the contributions of nurses. Acknowledging and valuing nursing privilege may enhance overall job satisfaction and retention among nursing professionals.

### 11.5 Patient‐centred care

The application of nursing privilege in patient care settings may result in more patient‐centred care. Nurses empowered with privileges are likely to contribute more actively to decision‐making processes, positively impacting the quality and outcomes of patient care.

### 11.6 Research and innovation

Researchers in nursing and healthcare may use the concept of nursing privilege as a framework for exploring new avenues of study. This could lead to innovative approaches in nursing practice, education and policy development.

### 11.7 Addressing disparities

An understanding of nursing privilege can shed light on potential disparities within the nursing profession. This awareness may prompt efforts to address and rectify any imbalances in access to resources, recognition or opportunities among nursing professionals.

### 11.8 Advocacy and empowerment

Nurses, armed with a clearer understanding of nursing privilege, may become advocates for their professional rights and contributions. This empowerment can lead to increased visibility, influence and recognition for the nursing profession.

### 11.9 Interprofessional collaboration

Recognizing nursing privilege may facilitate better collaboration among healthcare professionals. Understanding the unique contributions of nurses can foster collaborative decision‐making and a more cohesive approach to patient care.

### 11.10 Global perspectives

The concept of nursing privilege may be explored and adapted in different cultural and global contexts. This could lead to a more inclusive and globally relevant understanding of the role and privileges of nurses in diverse healthcare systems.

## 12 LIMITATIONS

This study, while providing valuable insights into the concept of ‘nursing privilege’, is not without limitations. First, the scope of the study may present constraints as it might not comprehensively cover all potential dimensions or variations of ‘nursing privilege’. This limitation could result in a partial understanding of the concept, overlooking certain crucial aspects that could contribute to a more holistic perspective. Additionally, the generalization of findings may be challenging due to variations in healthcare systems, cultural contexts and nursing practices, underscoring the need for cautious interpretation.

Secondly, there is a risk of subjectivity in the analysis of attributes and definitions. The interpretation of ‘nursing privilege’ may vary among individuals, introducing potential bias into the study. Furthermore, the study might be time‐sensitive, capturing the understanding of the concept at a specific point. Changes in healthcare policies, practices or societal attitudes over time may not be fully represented, limiting the study's temporal relevance.

Importantly, the interdisciplinary nature of the concept ‘privilege’ may pose challenges, as the study may not fully investigate into its cross‐disciplinary dimensions. Addressing these limitations is crucial for an unbiased and objective interpretation of the study's outcomes and for recognizing the potential constraints that may influence the understanding of ‘nursing privilege’ in diverse contexts.

## 13 CONCLUSION

In conclusion, fostering a profound comprehension to the concept of nursing privilege, is paramount for the advancement of the nursing profession. The concept of nursing privilege accentuates not only the autonomy but also the profound responsibilities shouldered by nurses. This depth of understanding serves as a catalyst for transformative improvements within the nursing domain. By clarifying and embracing the intricacies of nursing privileges, healthcare professionals are better equipped to communicate effectively, delineate and refine professional roles and foster efficient interprofessional collaboration.

This enhanced clarity of nursing privilege contributes to a more cohesive and synergistic healthcare environment. It empowers nurses to assert their roles confidently and advocate for optimal patient care. Furthermore, this comprehension facilitates a more informed and collaborative approach to healthcare decision‐making, breaking down silos within the healthcare system. Ultimately, the culmination of these efforts translates into tangible benefits for patients, as the delivery of care becomes more streamlined, efficient and of a consistently higher standard. In essence, a clear grasp of nursing privileges is not merely an academic pursuit; it is a catalyst for transformative improvements that resonate across the entire healthcare landscape.

## AUTHOR CONTRIBUTIONS

AAA and AJN made substantial contributions to the conception and design, or acquisition of data, or analysis and interpretation of data, involved in drafting the manuscript or revising it critically for important intellectual content, given final approval of the version to be published. Each author should have participated sufficiently in the work to take public responsibility for appropriate portions of the content and agreed to be accountable for all aspects of the work in ensuring that questions related to the accuracy or integrity of any part of the work are appropriately investigated and resolved.

## ACKNOWLEDGEMENTS

The publication of this article was funded by Qatar National Library. Hamad Medical Corporation Open Access publishing facilitated by the Qatar National Library, as part of the Wiley ‐ Qatar National Library agreement.

## CONFLICT OF INTEREST STATEMENT

The authors declare no conflicts of interest.

## ETHICS STATEMENT

None declared.

## Data Availability

None declared.
